# Percutaneous transluminal interventions of transplant renal artery stenosis: A case series study

**DOI:** 10.1016/j.amsu.2022.103563

**Published:** 2022-04-05

**Authors:** Amjad Ghareeb, Naji Alhamid, Qussai Hassan, Mohammed Ali Nahas

**Affiliations:** a Faculty of Medicine, Damascus University, Damascus, Syria; b Nephrology Department, Kidney Surgical Hospital in Damascus, Syria; c Chief of Nephrology Department and Kidney Transplant Unit, Al Assad University Hospital, Damascus University, Damascus, Syria; d Chief of Vascular and Endovascular Surgery Department, Al Assad University Hospital, Damascus University, Damascus, Syria

**Keywords:** Transplant renal artery stenosis, Percutaneous transluminal angioplasty, Endovascular intervention, Balloon angioplasty, Stent angioplasty, Renal graft transplantation

## Abstract

**Introduction:**

and importance: Transplant renal artery stenosis (TRAS) is a well-recognized vascular complication after kidney transplant that can lead to graft loss, when it is diagnosed early and treated appropriately it may prevent kidney damage and related systemic squeals.

**Case presentation:**

This case-series represents our center experience in managing TRAS using percutaneous transluminal angioplasty [either balloon angioplasty (PTA) or stent placement (PTAS)] in 11 patients.

**Clinical discussion:**

All treated patients experienced immediate total recovery of renal function and normalization of arterial blood pressure without any drug or reducing the number of drugs used; no complications related to the intervention were reported.

**Conclusion:**

PTA or PTAS of TRAS can be considered safe and effective when it diagnosed and treated early.

## Highlights

• Transplant renal artery stenosis is a vascular complication after kidney transplant that can lead to graft loss.
• When it is diagnosed and treated appropriately it prevents kidney damage and systemic sequelae.
• Endovascular intervention is the treatment of choice for Transplant renal artery stenosis or other arterial complications that may compromise the transplant kidney vitality.
• Percutaneous transluminal angioplasty either balloon angioplasty or stent placement is considered safe and effective.

## 1 Introduction

Kidney transplant is considered the treatment of choice for end-stage renal disease (ESRD) giving patients a good quality of life [1]. Transplant renal artery stenosis (TRAS) represents one of the major causes of graft failure with reported incidence between (1%–23%) representing about (85%) of vascular complications [2]. It usually occurs between three months and two years after renal transplantation, peaking in the first 6 months post-transplant with a higher incidence in elderly transplanted patients [3]. TRAS presents with many clinical manifestations like; refractory hypertension, oedema and allograft dysfunction in the absence of rejection [3]. Etiologically, TRAS is timely fashioned classified: early and late. The early one may occur due to anastomotic technical defects which need immediate correction or because of prolonged cold ischemia time during surgery which cause vascular wall damage and consequently late fibrosis (acute tubular necrosis), while late TRAS may occur due to trauma of the donor or recipient artery during surgery [4,5].

This retrospective case series represents 11 patients suffering from TRAS treated successfully with percutaneous transluminal angioplasty [either balloon angioplasty (PTA) or stent placement (PTAS)]. The study has been approved by the ethical committee of Damascus University.

## 2 Presentation of case

We present a retrospective case series of eleven male patients with an average age of 33.5 years old (table. 1), diagnosed as TRAS. The average time from transplantation to TRAS diagnosis was 21.2 months. This Study was conducted in Al Assad University Hospital, Damascus, Syria. All procedures were done by the professor and chair of department of vascular and endovascular surgery. The study was carried out in accordance with the Declaration of Helsinki.

**Table 1:** Patients information and the follow-ups.

Patient no.	Age (years)	Time from transplant to presentation (Months)	Symptoms and complaints upon presentation	Number of antihypertensive drugs before the intervention	Serum creatinine upon presentation (mg/dl)	Anastomosis	Site of lesion	Intervention	Stent/Balloon used	Number of antihypertensive drugs after the intervention	Follow up (Months)	Reevaluation after follow up (creatinine mg/dl)
1	30	48	HTN, CKD, CLLI	3	1.9	EE RIIA	Pre-anastomosis, Right common iliac artery (Total occlusion)	Recanalization and PTAS of RCIA	8 × 57 mm Stent **Visipro EV3®**	0	66	1
2	35	48	HTN, CKD	1	2.7	EE RIIA	anastomotic	PTA	6 × 14 mm Balloon **Brosmed Medical Co. LTD®**	0	43	0.9
3	37	48	HTN, CKD	4	2.2	ES REIA	anastomotic	PTA	5 × 18 mm Balloon **Hyppocampus Invatec®**	0	40	0.9
4	39	32	HTN	4	1.4	ES REIA	Anastomosis	PTAS	5.5 × 10 mm Stent **Dynamic renal, Biotronik®**	1	26	1.1
5	20	24	HTN, CKD	2	2	EE RIIA	anastomotic	PTAS	5 × 19 mm Stent **CID S·P.A®**	0	44	1
6	47	11	HTN	4	1.2	ES LCIA	Anastomosis	PTAS	5 × 12 mm Stent **Hyppocampus Invatec®**	1	26	1.1
7	44	7	HTN, CKD	2	2.1	EE RIIA	Anastomosis	PTAS	5.5 × 10 mm Stent	0	19	1.1
8	20	6	HTN	2	1.2	EE RIIA	Post anastomotic, Upper lobe artery	PTA	3 × 12 mm Balloon **Terumo, Hirya®**	1	56	0.8
9	41	5	HTN, CKD	2	2	ES RCIA	Post anastomotic	PTAS	6 × 12 mm Stent **Dynamic Renal, Biotronik®**	1	26	1.2
10	22	3	HTN	2	1.1	ES RCIA	anastomotic	PTAS	5 × 14 mm Stent **Paramount EV3®**	1	38	0.8
11	20	1	HTN	3	1.2	ES RCIA	Anastomosis	PTAS	6 × 10 mm Stent **Dynamic Renal, Biotronik®**	0	14	0.8

**Abbreviations:** HTN, hypertension; CKD, chronic kidney disease; CLLI, critical lower limb ischemia; EE, end to end; ES, end to side; RIIA, right internal iliac artery; REIA, right external iliac artery; RCIA, right common iliac artery; LCIA, left common iliac artery; PTA, percutaneous transluminal angioplasty; PTAS, PTA with stent.

The study has been reported in line with the PROCESS 2020 criteria. Our strategy aimed to confirm the clinical diagnosis of TRAS by color Doppler ultrasonography (CDU) followed by a selective digital subtraction angiography (DSA) with intention to treat stenotic lesions in the same session.

All patients had hypertension in their clinical complaints upon presentation while chronic kidney disease was observed in 6 patients (No. 1,2,3,5,7,9). Hemodynamic study focused on measuring systolic peak velocity (>200 cm/s), acceleration time in the transplant renal and intra-renal arteries (>0.15 s), ratio peak systolic velocity in the transplant renal artery –to – iliac donor artery (>1.8), documenting tardus-parvus wave in all transplanted kidneys.

The systolic peak velocity (SPV) was: (240–300 cm/s) at the anastomosis in eight patients, post anastomotic in two patients (No. 8,9) while in patient (No. 1) no peak systolic velocity augmentation was noticed because of total occlusion of the right common iliac artery (RCIA) (donor axis), this patient was suffering also from critical limb ischemia (CLI) in his right lower limb. The internal iliac artery (end to end anastomosis) represented the donor artery in five patients (No. 1,2,5,7,8), the common iliac artery (end to side anastomosis) in four patients (No. 6,9,10,11) while the external iliac artery (end to side anastomosis) was the donor in patients (No. 3,4).

**Fig. 1:**
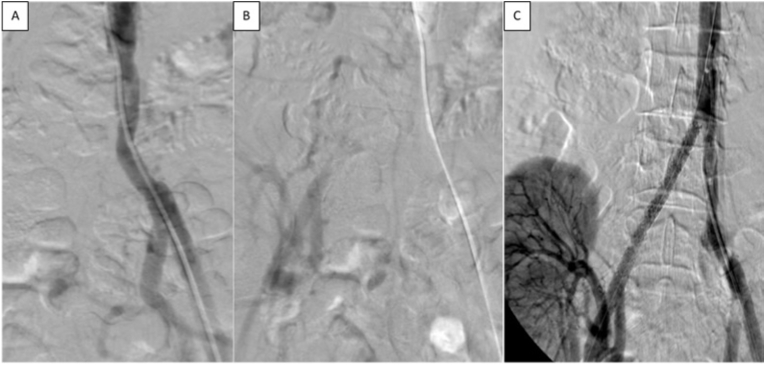
Patient No.1 **(A,B)**Total occlusion of the right common iliac artery, the transplanted kidney anastomosed to the right internal iliac artery. **(C)**Recanalization and stenting of the right common iliac artery.

A Well-controlled arterial pressure and an appropriate kidney preparation were obtained previously in all patients. Under local anesthesia, using an appropriate angiographic projection to reveal the lesion site, we documented a total right common iliac artery occlusion (pre-anastomotic) in one patient (No. 1) (Fig. 1) where the renal insufficiency was associated with (CLI) in his right lower limb, in patients (No. 8,9) a sever stenotic lesion was documented at the origin of the upper renal lobe artery and mid renal artery trunk respectively (post-anastomotic), while in the remaining eight patients the lesions were anastomotic.

**Fig. 2:**
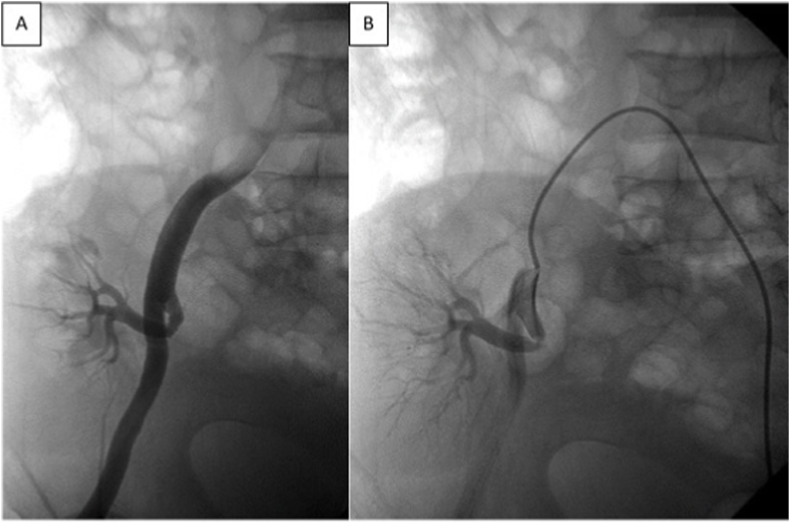
**(A)**Severe anastomotic stenosis. **(B)**Treated by PTAS.

**Fig. 3:**
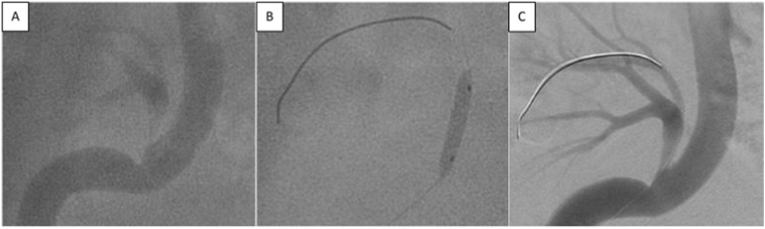
Patient No.3 **(A)**Severe stenosis of transplanted artery at the anastomosis with right external iliac artery. **(B)**Balloon angioplasty. **(C)**Transplanted renal artery after angioplasty.

The total common iliac occlusion patient (No.1) was crossed, through ipsilateral common femoral artery, by a hydrophilic 0,035 wire and 6F vert catheter and then stented, patients (No. 2,5,7,8) were approached through a contralateral femoral access, introducing an 8F guiding catheter engaging selectively the internal iliac artery (Fig. 2). The remaining patients were treated through an ipsilateral common femoral access using 7F sheath, no guiding catheter was used in this group of patients but a 6F vert catheter employed to engage the lesion. A 0.014 wire used to cross all the above lesions which were treated as mentioned in table (Fig. 3).

contrast injected was less than 75 ml in all procedures. In patient (No. 1) a bare metal stent was deployed directly after recanalization of the common iliac artery. While in the remaining cases a balloon angioplasty was done first then a bare metal stent was deployed in case of residual stenosis over 30% (table. 1).

All patients were given (300 mg) clopidogrel and (81 mg) aspirin the day before the procedure and (50 IU/kg) of heparin sodium was administered immediately before the procedure. (75 mg) clopidogrel, (81 mg) aspirin and (10 mg) atorvastatin once daily were continued life-long after discharge.

All patients were followed by clinical and lab evaluation including blood pressure measurement and serum creatinine monitoring, in addition to CDU which was done immediately after the procedure, before hospital discharge, at 1, 3, 6, 12 months in the first year, then twice yearly. SPV measurement and SC showed normal values. Blood pressure values became normal in six patients (No. 1,2,3,5,7,11). A single antihypertensive drug was used in the other five patients, the CDU study showed no complications related to the vascular procedures. After 60 months, the patient (No. 1) developed graft loss because of chronic rejection without any evidence of vascular complications documented by a new DSA, the patient was still free of lower limb ischemia with good pedal pulses.

## 3 Discussion

TRAS is considered as an important cause of allograft dysfunction and refractory hypertension following transplantation in (1–5%) of patients [6]. It can be diagnosed by computed tomography angiography, magnetic resonance angiography, CDU and DSA which remaines accepted as the golden standard for TRAS diagnosis [7]. Treatments modalities include endoluminal procedures (PTA/PTAS) and open surgery. The latter is considered as a rescue therapy in case of unsuccessful angioplasty or severe stenosis. However, open surgery may lead to many complications such as graft loss, ureteral injury and reoperation [2]. Endovascular intervention is preferred over surgery and may be considered the treatment of choice for TRAS because it is repeatable and has less complication like re-stenosis, renal artery injury or thromboembolism [8]. It is preferred for linear, short and proximal stenosis with success over 80%, higher technical success rates and clinical improvement are reported when PTAS has been used [[9], [10], [11]]. Indications for stent placement (PTAS) include: Residual stenosis after PTA of more than (30%), creation of flow limiting flap or presence of pressure gradient of more than (10 mm Hg) after PTA [2]. Clinical success rates following PTA, PTAS or combination were (65.6–94%) and technical success rates higher than (90%) [7]. Willicombe et al. [12] reported her findings regarding the role of donor specific antibodies in the development of post-anastomotic TRAS where patients with post-anastomotic TRAS were more likely to develop a rejection secondary to arteritis [OR: 4.83 (1.47–15.87), p¼0.0095] and capillaritis [OR: 3.03 (1.10–8.36), p¼0.033]. On the other hand, post-anastomotic TRAS were significantly more likely to develop de novo class II Donor Specific Antibodies compared to TRAS that occurs pre-anastomosis or at the anastomosis. These findings recommend possible screening for TRAS in patients who develop allograft dysfunction with de novo class II Donor Specific Antibodies. A systematic review by A. T. Ngo et al. [7] supports PTA/PTAS procedures in TRAS which were done in our cases. The follow-ups in our cases showed good results. However, some needed one antihypertension drug, but they had normal creatinine range. More detailed follow-ups were not possible due to the financial and social hurdles facing Syrians at the moment [13]. These unique circumstances have put additional pressure on hospitals as only few major one remained functional during the war [14].

## 4 Conclusion

PTA and PTAS is a safe, effective and repeatable treatment modality in managing TRAS when it is diagnosed early. Many causes of TRAS are explained and they are linked to their time manner. The role of Donor Specific Antibodies in the pathogenesis of TRAS in post-transplant period are goals for further researches.

The case has not been presented at a conference or regional meeting.

## Consent of patient

Written informed consent was obtained from the patient for publication of this case report and accompanying images.

A copy of the written consent is available for review by the Editor-in-Chief of this journal on request.

## Ethical approval

Research studies involving patients require ethical approval. Please state whether approval or exemption has been given, name the relevant ethics committee and the state the reference number for their judgement. Please give a statement regarding ethnical approval that will be included in the publication of your article, if the study is exempt from ethnical approval in your institution please state this.

This study has been approved by the ethical committee of Damascus University.

## Sources of funding

All sources of funding should be declared as an acknowledgement at the end of the text. Authors should declare the role of study sponsors, if any, in the collection, analysis and interpretation of data; in the writing of the manuscript; and in the decision to submit the manuscript for publication. If the study sponsors had no such involvement, the authors should so state.

We received no funding in any form.

## Authors contribution

Please specify the contribution of each author to the paper, e.g. study concept or design, data collection, data analysis or interpretation, writing the paper, others, who have contributed in other ways, should be listed as contributors.

AG: Data curation; Investigation; Formal analysis; Writing - review & editing contributions; Software; Methodology. NA: Data curation; Investigation; Resources; Writing - review & editing contributions; Methodology. QH: Supervision; Methodology; Validation. MAN: Conceptualization; Project administration; Supervision; Formal analysis; Validation.

## Guarantor

The Guarantor is the one or more people who accept full responsibility for the work and/or the conduct of the study, had access to the data, and controlled the decision to publish.

Amjad Ghareeb is the Guarantor.

## International journal of surgery Case Reports

The following information is required for submission. Please note that failure to respond to these questions/statements will mean your submission will be returned. If you have nothing to declare in any of these categories, then this should be stated.

## Consent

Studies on patients or volunteers require ethics committee approval and fully informed written consent which should be documented in the paper.

Authors must obtain written and signed consent to publish a case report from the patient (or, where applicable, the patient's guardian or next of kin) prior to submission. We ask Authors to confirm as part of the submission process that such consent has been obtained, and the manuscript must include a statement to this effect in a consent section at the end of the manuscript, as follows: "Written informed consent was obtained from the patient for publication of this case report and accompanying images. A copy of the written consent is available for review by the Editor-in-Chief of this journal on request”.

Patients have a right to privacy. Patients’ and volunteers' names, initials, or hospital numbers should not be used. Images of patients or volunteers should not be used unless the information is essential for scientific purposes and explicit permission has been given as part of the consent. If such consent is made subject to any conditions, the Editor in Chief must be made aware of all such conditions.

Even where consent has been given, identifying details should be omitted if they are not essential. If identifying characteristics are altered to protect anonymity, such as in genetic pedigrees, authors should provide assurance that alterations do not distort scientific meaning and editors should so note.

All patients consented to the use of case details and images for scientific purposes.

## Registration of research studies

In accordance with the Declaration of Helsinki 2013, the Editors of IJS Case Reports require that reports that are ‘First in Man’ studies should be registered prospectively and failing that retrospectively. There are many places to register your First in Man case report:

• Clinicaltrials.gov – for all human studies – free
• Chinese Clinical Trial Registry chictr.org.cn – for all human studies - free
• Researchregistry.com – for all human studies – charge • ISRCTN.com – for all human studies – charge
• There are many national registries approved by the UN that can be found here

Elsevier does not support or endorse any registry.

1. Name of the registry: Researchregistry.com
2. Unique identifying number or registration ID: researchregistry7547
3. Hyperlink to your specific registration (must be publicly accessible and will be checked):
https://www.researchregistry.com/register-now#home/registrationdetails/61e1a652652dcd001e77aa9b/

## Declaration of competing interest

All authors must disclose any financial and personal relationships with other people or organisations that could inappropriately influence (bias) their work. Examples of potential conflicts of interest include employment, consultancies, stock ownership, honoraria, paid expert testimony, patent applications/registrations, and grants or other funding.

The authors have no conflict of interest to declare.
